# All in One, Self‐Powered Bionic Artificial Nerve Based on a Triboelectric Nanogenerator

**DOI:** 10.1002/advs.202004727

**Published:** 2021-05-03

**Authors:** Qian Zhang, Zixuan Zhang, Qijie Liang, Qiongfeng Shi, Minglu Zhu, Chengkuo Lee

**Affiliations:** ^1^ Department of Electrical and Computer Engineering National University of Singapore 4 Engineering Drive 3 Singapore 117576 Singapore; ^2^ Center for Intelligent Sensors and MEMS (CISM) National University of Singapore 5 Engineering Drive 1 Singapore 117608 Singapore; ^3^ National University of Singapore Suzhou Research Institute (NUSRI) Suzhou Industrial Park Suzhou 215123 China; ^4^ Department of Physics National University of Singapore 2 Science Drive 3 Singapore 117551 Singapore; ^5^ Singapore Institute of Manufacturing Technology and National University of Singapore (SIMTech‐NUS) Joint Lab on Large‐area Flexible Hybrid Electronics National University of Singapore 4 Engineering Drive 3 Singapore 117576 Singapore; ^6^ NUS Graduate School for Integrative Science and Engineering (NGS) National University of Singapore Singapore 117456 Singapore

**Keywords:** bionic artificial nerves, nervous system, self‐powered sensors, sensory system, triboelectric nanogenerators

## Abstract

Sensory and nerve systems play important role in mediating the interactions with the world. The pursuit of neuromorphic computing has inspired innovations in artificial sensory and nervous systems. Here, an all‐in‐one, tailorable artificial perception, and transmission nerve (APTN) was developed for mimicking the biological sensory and nervous ability to detect and transmit the location information of mechanical stimulation. The APTN shows excellent reliability with a single triboelectric electrode for the detection of multiple pixels, by employing a gradient thickness dielectric layer and a grid surface structure. The sliding mode is used on the APTN to eliminate the amplitude influence of output signal, such as force, interlayer distance. By tailoring the geometry, an L‐shaped APTN is demonstrated for the application of single‐electrode bionic artificial nerve for 2D detection. In addition, an APTN based prosthetic arm is also fabricated to biomimetically identify and transmit the stimuli location signal to pattern the feedback. With features of low‐cost, easy installation, and good flexibility, the APTN renders as a promising artificial sensory and nervous system for artificial intelligence, human–machine interface, and robotics applications.

## Graphical abstract

An all‐in‐one, tailorable artificial perception, and transmission nerve is developed for mimicking the biological sensory and nervous ability to detect and transmit the location information of mechanical stimulation. The as‐mentioned process consumes no electrical energy at all. In addition to detecting 2D stimuli by a single‐electrode mode, a self‐powered prosthetic arm is also demonstrated.

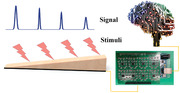

## 1 Introduction

One of the most exciting developments of the past decades is the discovery of sensory and nervous systems in our body that play important roles to control and receive feedback from limbs.^[^ ^1^, ^2^, ^3^ ^]^ Biologically, the sensory systems associated with senses convert stimuli (such as force, light, or sound) into electrical signals in the nervous systems, and then the electrical activities will be interpreted by the brain as stimuli.^[^ ^4^, ^5^, ^6^, ^7^ ^]^ With the development of Von Neumann‐based computing systems, artificial sensory and nervous systems biomimicking the biological systems have attracted many attentions due to its inspirer applications, such as artificial afferent nerve used in prosthetic limb,^[^ ^8^ ^]^ construct animal–robot interaction,^[^ ^9^ ^]^ build feedback of artificial intelligence.^[^ ^10^ ^]^ Although great achievements have been made, most reported artificial sensory and nervous systems exhibit complicated structures, intricate interconnections, and high energy consumptions.^[^ ^11^ ^]^ Pursuing low, even zero‐energy consumption as well as simple structure of artificial sensory and nervous systems still faces challenges. In fact, there have been a lot of works focusing on each of the three main functional elements (bionic sensor, synaptic transistor, signal cable) of the artificial sensory and nervous systems in the past few years.^[^ ^12^, ^13^, ^14^, ^15^, ^16^ ^]^ Thus, the interference between two different elements may be induced in the artificial sensory and nervous system. Thereby, an artificial sensory and nervous system with an all‐in‐one structure is undoubtedly a better choice for reducing the interference in signal transmission.

Since its first invention, triboelectric nanogenerator (TENG) has been proven as a promising energy harvesting technology, which receives rapid development as diverse energy harvesters and self‐powered sensors globally.^[^ ^17^, ^18^, ^19^, ^20^, ^21^, ^22^, ^23^, ^24^, ^25^, ^26^ ^]^ By directly converting mechanical stimuli to electrical signals, TENGs can operate as self‐powered sensors for tactile sensing without extra power supply, which is vital for developing maintenance‐free systems.^[^ ^27^, ^28^, ^29^, ^30^, ^31^, ^32^ ^]^ Therefore, TENG technology provides an ideal approach to realize artificial nerve with self‐powered capability,^[^ ^33^, ^34^ ^]^ because of its diverse and simple device configuration,^[^ ^35^, ^36^, ^37^ ^]^ ultrawide material applicability,^[^ ^38^, ^39^, ^40^ ^]^ flexibility/stretchability,^[^ ^41^, ^42^, ^43^ ^]^ and low‐cost.^[^ ^44^, ^45^, ^46^ ^]^ There are some artificial neural systems based on TENG focusing on mimicking biological systems to detect amplitude of force,^[^ ^47^, ^48^ ^]^ frequency of mechanical stimulation,^[^ ^49^ ^]^ and rotational movement.^[^ ^50^ ^]^ However, few of them detect location of external mechanical stimuli, such as finger or object touch. On the other hand, the artificial sensory used in many nervous systems based on TENG exhibit large number of electrodes for multipixels monitoring. Although these developed pressing‐type artificial sensory exhibit relatively stable and high output performance, they require the designs of complicated structures and many electrodes for different pixels.^[^ ^51^, ^52^, ^53^, ^54^, ^55^ ^]^ To overcome this issue, multiple distinguishable output signals can be introduced into a single‐electrode TENG, then forming one electrode sensory for more pixels.^[^ ^56^, ^57^ ^]^ When an object in different positions in the triboelectric series touches on the surface of a single‐electrode TENG, positive and negative charges are generated on the two dielectric surfaces of the object and TENG, respectively. During the approaching process, a different amount of charge is forced to flow from the TENG electrode terminal to the ground, thus generating an output signal. It is found and proven that the output performance of TENG is influenced by the thickness of its dielectric material.^[^ ^58^, ^59^, ^60^, ^61^ ^]^ However, the presence of factors such as force and interlayer distance reduce its reliability and there are few works that explore the application of this property.

In this work, we present a tailorable, TENG‐based artificial perception and transmission nerve (APTN) with an all‐in‐one structure, which converts mechanical stimulus to an electrical neural‐stimulating signal, then transmitting the mechanosensitive signal to a driver circuit. Then the APTN transmits the mechanosensitive signal to the driver circuit. Thickness difference in dielectric layer of the APTN is investigated to achieve distinguishable output signal for multiple pixels with single‐electrode output. Then, the location of mechanical stimulation on the APTN is recognized by analysing the signal magnitude. A grid structure is fabricated on APTN surface to control output performance factors of TENG. The as‐mentioned process of signal generation and transmission consumes no electric energy at all. The tailorable APTN can be designed into a wide variety of prototype devices as needed, such as an L‐shaped device to further recognize the location of 2D mechanical stimulation without requiring the integration of multiple sensing units. Subsequently, the APTN is designed as a prosthetic arm to demonstrate multifunctional touch interaction for human–machine hybrid perceptual enhancement, inspired by the human arm in a biological system. This artificial sensory and nervous system will be a promising technology for various applications in bioinspired electronics, such as restore touch perception of disabled persons, artificial intelligence, human–machine interface, and so on.

## 2 Results and Discussions

**Figure 1:**
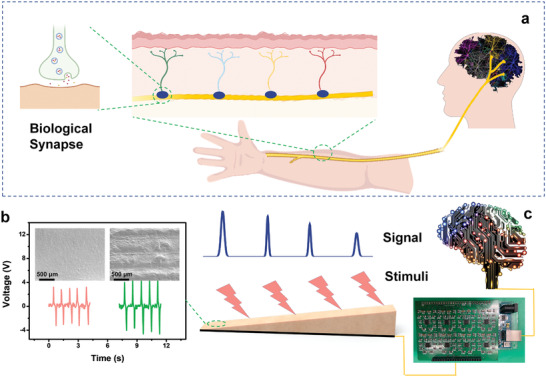
Schematic of APTN compared with human sensory neurons. a) A biological afferent nerve that is mechanical stimulated. b) Voltage and SEM image of APTN with and without microstructure on surface. c) Schematic of the function of APTN.

Biologically, the externally applied mechanical stimulus is converted into receptor potentials by mechanoreceptors. The receptor potentials are encoded by synapses, which are formed between the multiple afferent neurons and the interneuron in the spinal cord, and then awaken the interneurons. Subsequently, the encoded postsynaptic potentials are transmitted to the cortex in turn through the interneurons to recognize the location of the external mechanical stimulation (**Figure** **1**a). The APTN in this work mimicking perception and transmission nerve detects and recognizes the location of mechanical simulation in real‐time. We proposed a new strategy here by constructing the dielectric layer with gradient thickness to detect the location of the external mechanical stimulation. Moreover, the APTN can also transmit mechanosensitive signal like a signal cable. The APTN was prepared by a simple fabrication process. As shown in Figure S1 (Supporting Information), a series of 3D‐printed molds for different gradient dielectric layers of APTN were prepared. The mixture of solutions A and B of silicone rubber was poured into the 3D‐printed mold to control the gradient of dielectric layer of APTN. Then, a conductive fabric is covered on silicone rubber as an electrode. The APTN was peeled off from the mold after the silicone rubber solidifying. The details are described in the Experimental Section. The as‐fabricated APTN is based on the single‐electrode TENG. By duplicating and transferring the microstructure of the 3D‐printed mold, the surface of APTN was patterned with microstructure. The microstructure on APTN surface was acquired with a uniform width of around 200 µm, as depicted in the scanning electron microscopy (SEM) image of Figure 1b. Comparing the output of APTN with a plain surface, the microstructure promotes the triboelectric effect by increasing the effective contact area and friction during contact/separation between silicone rubber and other materials. When touching on the surface of APTN, the mechanical stimulation is converted into potentials by the triboelectric effect (Figure 1c), which consumes no external energy. By connecting APTN and computer with a driver circuit, the tribo‐effect generated potential of APTN was transferred to the digital value. Subsequently, the digital value is transmitted and analyzed by a computer to recognize the location of the external mechanical stimulation.

**Figure 2:**
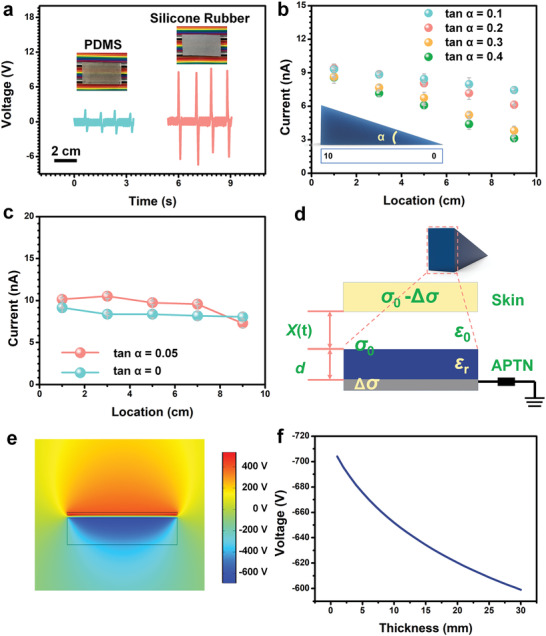
Performance and finite element simulation of the APTN. a) Comparison of triboelectric output voltage of APTN made by PDMS and silicone rubber. Inset is photographs of APTN (tan *α* = 0) made by different materials. b) Relationship between the location of mechanical stimulation and the current of the APTN with different gradient (tan *α* = 0.1, 0.2, 0.3, 0.4). c) The current of APTN with smaller gradient (tan *α* = 0, 0.05). d) The model of APTN for the calculation (friction object is skin). e) Finite element simulation of the potential distribution in the APTN and friction object. f) The decrease of electrode potential of APTN is a result of thickness of dielectric layer of APTN increase.

Silicone rubber is chosen here not only because it is biocompatible,^[^ ^62^ ^]^ but also the silicone rubber is positioned in the extreme negative end in the triboelectric series.^[^ ^63^ ^]^ As shown in **Figure** **2**a, the output of silicone rubber is much larger than that of Polydimethylsiloxane (PDMS) with the same thickness of 0.5 cm, which is preferable for conducting accurate sensing. This is because the magnitude of the voltage signals is used to distinguish the position of stimuli. The silicone rubber with larger voltage signals has a greater difference between two different positions with the same distance than PDMS. Therefore, the influencing factors of position distinguishment, such as force could be reduced. On the other hand, the signal‐to‐noise ratio could be calculated by dividing the signal voltage with noise voltage. The signal‐to‐noise ratio of PDMS is about 4, which is much poorer than that of the silicone rubber (11.54). The silicone rubber with larger signal‐to‐noise ratio also benefits for the signal analysis in the future applications.

The thicker edges of dielectric layers were changed from 0.5, 1, 2, 3, to 4 cm, while the thinner edges keep being 0 cm. So, 5 devices with different gradient (tan *α* = 0.05, 0.1, 0.2, 0.3, 0.4) were fabricated. The device with the same thickness of 0.5 cm (tan *α* = 0) was also fabricated for comparison. As shown in Figure 2b, with the location of mechanical stimulation changes from 1 to 9 cm, the output current decreases as the thickness of dielectric material increasing when the tan *α* is ranging from 0.1 to 0.4. Besides, the output current points aligned with a steeper line when the tan *α* increases from 0.1 to 0.4, which means a higher output current is generated by touching on the same location with the thinner dielectric film. There are no obvious changes in the output of APTEN with the tan *α* = 0.05 and 0, which has a small thickness difference in dielectric layer (Figure 2c). A cross‐section structure of the APTN with a fixed thickness dielectric material (*d*) is modeled in Figure 2d for theoretical analysis. The skin is used to be the stimulus object. The output‐thickness relationship of a single‐mode TENG based APTN is given by^[^ ^64^ ^]^

(1) $$V=\frac{\left(\sigma_{0}-\Delta \sigma \right)x\left(t\right)}{\varepsilon_{0}}-\frac{\Delta \sigma d}{\varepsilon_{0}\varepsilon_{r}}$$

Where *ε*_0_, *ε*_r_, *σ*_0_, and Δ*σ* are the vacuum permittivity, relative permittivity of the dielectric material, triboelectric charge density on the dielectric material, and transferred charge density on the electrode in a stage, and *x*(*t*) and *t* are the interlayer distance and time, respectively. The electric potential distribution in the TENG based APTN and the charge transfer between the electrode and the ground can be verified through numerical simulation using COMSOL. The proposed model is also shown in Figure 2d. The triboelectric charge density on the dielectric material was assumed to be −10 µC m^−2^. The electrode of APTN was connected to the ground. Figure 2f depicts the calculated results of the electric potential distribution in the APTN under different thicknesses of dielectric layer with different locations. When the APTN with a dielectric layer thickness of 1 mm, the electric potential is up to −703 V. It can be clearly seen that the electric potential on the electrode of APTN decreases dramatically with increasing thickness of dielectric material of APTN from 1 to 30 mm. In the simulation case, we only considered the influence of the electrostatic field on the electric potential, while the external factors such as surface charge distribution, device structure, resistance, and applied force were ignored. Therefore, there is a nonlinear relationship between the electric potential and thickness, which has some differences from the result of experiment. However, the simulation result shows that the electric potential increases with the increase of the thickness, which is an evidence that by analyzing the magnitude of the response output, the APTN could recognize the location of the mechanical stimulation. In addition, analogising to the function of the interneuron in the spinal cord, the proposed APTN was limited to transmitting and recognizing one location at a time. The APTN still lack the capability of recognizing simultaneous touching at multiple positions because of the working mechanism.

**Figure 3:**
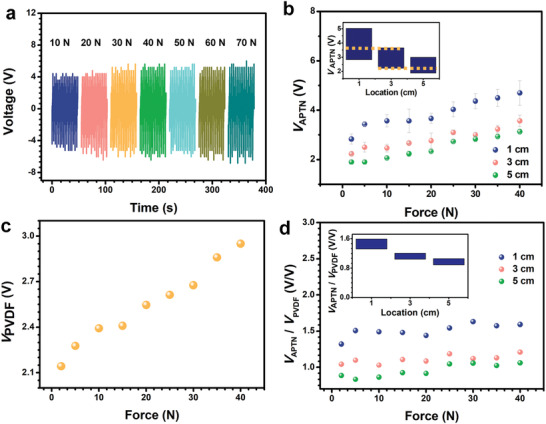
Performance of pressing on APTN under different forces. a) Voltage of APTN (*V*_APTN_) with a location of 5 cm, under different forces (tan *α* = 0.3). b) Relationship between the force and the voltage when stimulating on different locations of the APTN. Inset is column chart to compare the voltage of the APTN with different locations clearly. c) Voltage of PVDF (*V*_PVDF_) under different forces. d) *V*_APTN_ divided by output *V*_PVDF_.

Contact pressure is an essential element to impact on the output of TENG.^[^ ^65^, ^66^ ^]^ In order to test the relationship of output voltage, contact pressure, and thickness of dielectric layer accurately, a force gauge testing system is used to replace hand for output characterization. As shown in **Figure** **3**a, when the stage moving speed is fixed at 900 mm min^−1^, it can be seen that the voltage of APTN increases with force enhancing from 10 to 40 N. There is no obvious increment when the force increases from 50 to 70 N. The stability and durability measurement of the APTN is also tested by using a force gauge stage continuously touches on its surface for 1000 cycles (Figure S2, Supporting Information). The stage moving speed is fixed at 900 mm min^−1^, while the force is fixed at 20 N. In addition, the touched surface is kept to the location 1 cm. This device exhibited good stability, which make it reliable for potential applications. Figure 3b illustrates the peak voltage (*V*_APTN_) generated by APTN when the pressure was applied on different locations (1, 3, and 5 cm) of APTN. As shown in the inset of Figure 3b, the force changing from 2 to 40 N, which is in a large range, will influence the recognition of location. To eliminate the influence of force on location detection, a Poly(vinylidene fluoride) (PVDF) film was attached to the bottom of APTN. Figure S3 (Supporting Information) shows the responses of the APTN under various mechanical stimulus frequencies ranging from 0.20 to 1.90 Hz for the same gradient dielectric layers of APTN (tan *α* = 0.3) and position (5 cm). The responses are stable for all five frequencies during the four cycles. Besides, the peak voltage of the APTN increases with the frequency. When the APTN is touched multiple times, the value of frequency can be calculated through the time between interval peak voltages. Then the corresponding position can be found by analysis and judgment. In the case that the APTN is touched once, the frequency can be calculated by the duration of the crest. The higher the frequency, the narrower the crest, so as to find the corresponding position.

Figure 3c shows the relationship of Voltage from PVDF (*V*_PVDF_) and the applied force ranging from 2 to 40 N. It can be seen that *V*_PVDF_ linearly increases with applied force. When applied force is 2 N, the *V*_PVDF_ is 2.1 V. When applied force increasing to 40 N, *V*_PVDF_ increases to 2.9 V. Sensitivity of the PVDF is 0.01 N^−1^ based on the following Equation

(2) $$S=\frac{d\left(\Delta V_{\mathrm{PVDF}}/V_{\mathrm{PVDF}}\right)}{d\Delta F}$$

Where *S* is the sensitivity, Δ*V*_PVDF_ is voltage increment, and *V*_PVDF_ is the original voltage when force is applied, and Δ*F* is the force increment. To eliminate the influence of force, the *V*_APTN_ is divided by *V*_PVDF_. As shown in Figure 3d, the values of *V*_APTN_/*V*_PVDF_ of different locations are different after eliminating the influence of force by PVDF. Therefore, a large range of force influences the detection of APTN location. The method of attaching PVDF to the bottom of APTN may not only induce interference between two different elements but also increase cost in application. On the other hand, press‐separating on APTN requires an air gap and the performance is largely affected by the interlayer distance, which is difficult to control. To this end, a sliding model and grid structure on the APTN surface was introduced in application to keep a small range force and the same interlayer distance.^[^ ^67^ ^]^

**Figure 4:**
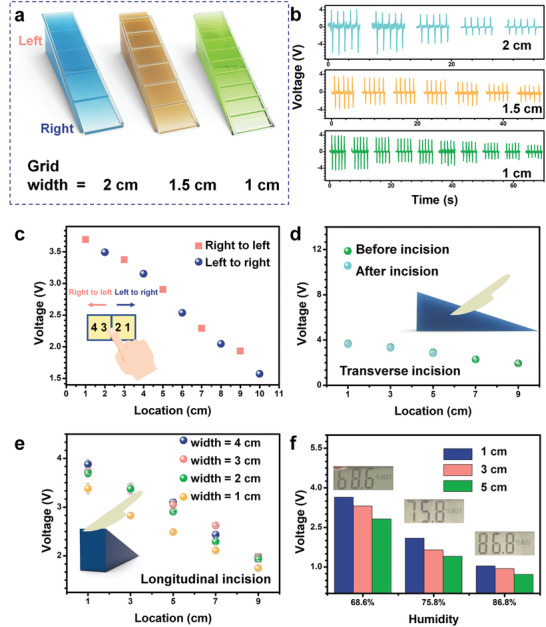
The resolution and tailorable performance of sliding on APTN. a) Schematic diagram of APTN with different grid width (2, 1.5, and 1 cm). b) Output voltage of APTN with different grid width. c) Output voltage of APTN under different slides directions on grids. Comparison of output voltage of APTN before and after d) transverse and e) longitudinal incisions to demo tailorable property of APTN. f) Tests of the APTN under different humilities.

**Figure** **4**a shows the grid with different width distances (2, 1.5, 1 cm) on the surface of APTN schematically. The grid made up of resin is deposited on the dielectric layer by 3D printing. Hight of the grid is fixed to be 1.5 mm. Sliding from right (thinner edge) to left side (thicker edge) of APTN with a finger to keep the *x*(*t*) in Equation (1) as constant. Because of the restriction of the grid, the finger contact points with silicone rubber are mainly concentrated in the centre area of every single grid. In addition, by using this sliding mode, force changes in a small range. The output voltage of APTN with different width of grids under sliding from right to left with a finger is shown in Figure 4b. The finger sliding on each of grids is repeated for 5 times. The voltage of APTN decreases when the sliding location changing from a thinner dielectric layer to a thicker dielectric layer. After 5 cycles of finger sliding, the responses are stable, revealing the excellent reliability of APTN for application. It could be also noted that the resolution of APTN is higher at a smaller width of the grid. For example, with 1 cm width of grid, the APTN can detect location with a small distance (location = 1, 2, 3, 4, 5, 6, 7, 8, 9, 10 cm), while the 2 cm width of grid can only detect a larger distance (location = 1, 3, 5, 7, 9 cm). During the sliding mode test, the output voltage depends on the sliding direction. Furthermore, the response time (*T*r) was examined by analysing the magnified detailed waveforms of the APTN with 2 cm width of grid (Figure S4, Supporting Information). A high sensitivity with a fast *T*r of 130 ms justifies the practicability of APTN for detecting position, which is of paramount importance for various further applications, such as robotic control. When finger is sliding from the right side to the left side on APTN, in which the detected location is 1, 3, 5, 7, 9 cm (pink color in Figure 4c), larger output is generated than sliding from left to right (detected location is 2, 4, 6, 8, 10 cm, blue color in Figure 4c). The contact pressure of the finger is controlled by controlling the speed of finger sliding. A metronome is used to control the speed. In addition, a force sensor is also used to measure the contact pressure. The results that generated from sliding a finger on the APTN in different directions (from left to right and from right to left) are only recorded when the force is 2 N. The dependence of the output of the force sensor on force is shown in Figure S5 (Supporting Information).

Biologically, sensory and nervous systems injury by mechanical damage is a serious health problem that affects many trauma patients.^[^ ^68^ ^]^ Researchers make a lot of effects to develop various strategies for better recovery of nerve functions. The tailorable property is not only important but also necessary for the artificial sensory and nervous systems to resist mechanical damage including being cut, pierced, and torn in real application.^[^ ^41^ ^]^ To investigate the tailorable property of the APTN, transverse and longitudinal incisions were made on the 10 cm long and 4 cm wide device. The schematic diagram of APTN with a transverse incision is shown inset in Figure 4d. A knife was used to make the incision. After the incisions, the APTN could still function. The peak output voltage of APTN remains the same before and after the transverse incisions at three different locations (1, 3, 5 cm). Subsequently, the output performances of the APTN before and after longitudinal incisions were also investigated and shown in Figure 4e. The direction of the incisions is also shown in the inset of Figure 4e. The average peak voltage of APTN before and after longitudinal incisions making on 2 and 3 cm are approximately the same. However, the average peak voltage decreased after making the longitudinal incision on 1 cm. This is because of the decreasing contact area between finger (about 1.5 cm wide) and 1 cm wide APTN. The APTN presents stable output signal before and after being incisions, which shows its advantageous of reliability in future application.

In order to check the influence of humidity on the output of APTN, the tests are conducted under different environments as shown in Figure 4f. The voltage amplitude varies with the change of humidity. The spray device is used to change the humidity on the surface of the device. Three groups of experiments are carried out under different humilities. The measured values of surface humidity of the 3 groups are shown in the inset of Figure 4f as 68.6%, 75.8%, 86.8%, respectively. In each group, the voltages of 3 locations on APTN are measured by sliding on the center point of the 1, 3, 5 cm grid. As illustrated in Figure S6 (Supporting Information), the output voltage as a function of humidity has a linear fitting and the fitting equation for the output voltage (y) of APTN with the location of 1, 3, and 5 cm, and the humidity (*x*) can be represented as *y* = 14.26–15.47x, *y* = 14.26–15.47x, *y* = 13.00–14.58x with the R‐squared value of 0.9667, 0.9544, 0.9530, respectively. The relationship between the output voltage and the humidity could suggest the baseline of the APTN under different humidifies. With the increase of humidity, the output voltage of the electrode will decrease significantly, but the location with thinner dielectric layer still shows a bigger voltage. This is because the presence of water will improve the dielectric constants of air and dielectric materials, causing a linear increase of effective dielectric permittivity. Thus, according to Equation (1), the output voltage of APTN decreases due to the increasing of effective dielectric permittivity.

**Figure 5:**
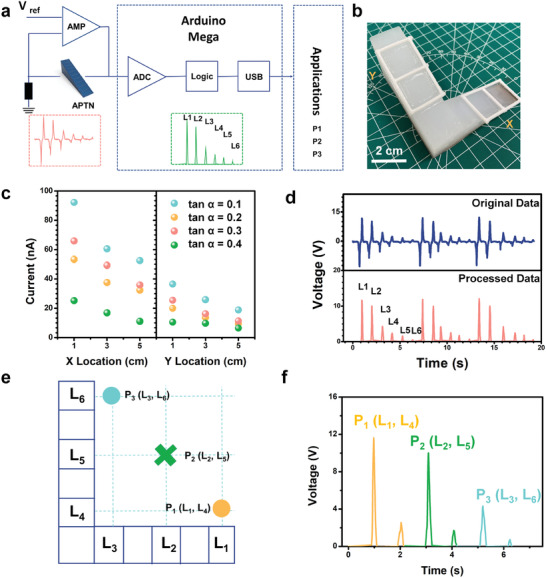
Performance and characteristic of the L‐shaped APTN. a) Circuit schematic to drive the APTN for applications. b) Photograph of the L‐shaped APTN. Scale bar is 2 cm. c) Output current of L‐shaped APTN with different thickness gradient. d) Original data and processed data of six different locations of the sliding stimuli (repeat 3 times). e) An L‐shaped APTN used for controlling the position of a tic‐tac‐toe game in a 2D plane. The *x* axis controlled the horizontal movement of the tic‐tac‐toe game and the *y* axis controlled the movement of the tic‐tac‐toe game on the vertical axis. f) Change in the voltage and achieve different positions when sliding on different grids.

A driver circuit was designed to communicate the APTN with a computer. **Figure** **5**a shows the circuit schematic to drive the APTN. The first stage was that the voltage of APTN was converted into the digital domain by an analogy to digital converter. This convertor was composed of an operational amplifier with a feedback resistor network. The output voltage was proportional to the peak voltage of APTN. The analogy output of the driver circuit was digitalized and synchronized by a microcontroller development board (Arduino), which integrated a low‐power microcontroller chip (Mega) as its central processor. The microcontroller development board had a total number of 20 internal on‐chip analogy‐to‐digital converters (ADC), and one of them was connected to the analogy output voltage as its input sensing signal. A power source (5 V) was used to power the Arduino board, while the APTN was connected to the Arduino board to provide the analogy output voltage as the input sensing signal. The current consumption of the board is 11.85 mA. So the energy consumption of the system is calculated by current multiply voltage is 59.25 mW. There was a lookup table built inside the microcontroller so as to map the digital value to the location of the mechanical stimulation. Furthermore, there was a built‐in universal‐serial‐bus interface on this development board, which enabled the microcontroller to communicate with personal computers. Based on the digital value of the output voltage, this development board could send corresponding commands to the personal computer side. Since there would be customized applications developed on the computer side, actions would be performed as the responses based on the received command. With the development of microchips nowadays, wearable/portable central processors with small size were reported.^[^ ^69^, ^70^ ^]^ Then the central processor of the artificial sensory and nervous system can be minimized and attached on or implanted in the APTN in the future. The size of the artificial sensory and nervous system will only depend on the size of APTN. From Figure 4f, we can find the humidity will influence the voltage output of APTN. Thus, a denoising process is necessary by baseline tracking processing. Figure 5b shows the optical image of the APTN designed in L‐shape. This L‐shaped APTN is with a single‐electrode and can further recognize the 2D location of external mechanical stimulation without requiring the integration of multiple sensing units. It should be noted that the single‐electrode L‐shaped APTN only needs one channel so that contributed to simplify the signal processing. To investigate the best gradient of the L‐shaped device, the output peak current of APTNs with different gradients (tan *α* = 0.1, 0.2, 0.3, 0.4) was fabricated and measured. A series of 3D‐printed moulds with different gradient was designed and fabricated at first (Figure S7, Supporting Information). As shown in Figure 5c, the output current of L‐shaped APTN decreases as the thickness of dielectric material increases. The gradient of output current increases when the tan *α* of L‐shaped APTN changes from 0.1 to 0.4, which is consistent with Figure 2b. Considering both magnitude and gradient of output, the L‐shaped APTN with a gradient of tan *α* = 0.3 was chosen to connect with the computer. When a finger was sliding on a certain grid, the voltage of APTN was quickly generated and transferred to the digital value. As shown in Figure 5d, the original voltage data of APTN by once sliding has a positive peak and a negative peak while the processed data only has a positive peak. Six different locations of the sliding were stimulated (repeat 3 times). The processed peak voltage values of different grids were different from each other so that the microcontroller could easily identify them. By using this shape, the APTN can be used to play the classic Tic‐Tac‐Toe game (also called Noughts and Crosses). As shown in Figure 5e, both *x* and *y*‐axis of the L‐shaped APTN have 6 grids, naming as L_1_, L_2_, L_3_, L_4_, L_5_, L_6_. Each two of the grids on a different axis controls the positions of Noughts or Crosses. For example, when a finger sliding on L_1_ and L_4_, the output voltage of the L‐shaped APTN would be generated and transmitted to the driver circuit. Then, the peak circuit is transferred. According to this peak voltage, the microcontroller would send the machine‐codes that confirm the position to be Position 1 (P1). Subsequently, when the finger slides on L_2_ and L_5_, the position will be moved to P2. As shown in Figure 5f, the three positions (P_1_ (L_1_, L_4_), P_2_ (L_2_, L_5_), P_3_ (L_3_, L_6_)) peak voltages of the driver circuit are (11.6, 2.5 V), (10.02, 1.71 V), (4.3, 0.73 V).

**Figure 6:**
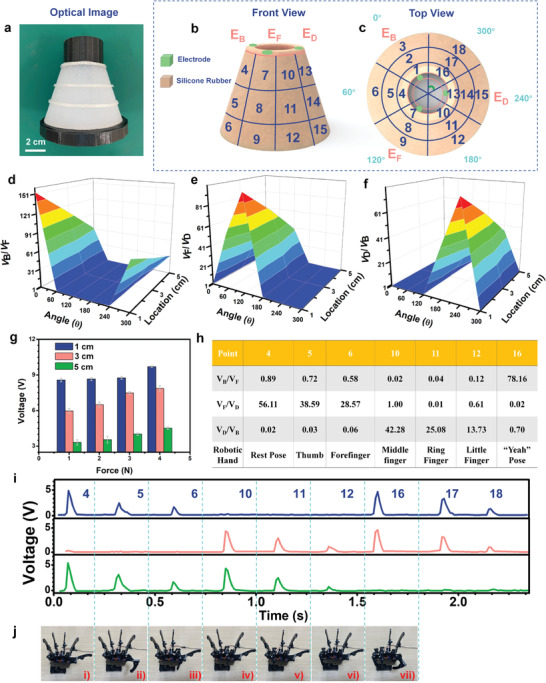
Performance and characteristic of the APTN based prosthetic arm. a) Photograph of APTN based prosthetic arm. Scale bar is 2 cm. b) Front view and c) Top view of the APTN based prosthetic arm with 3 electrodes (*E*_B_, *E*_D_, *E*_F_). Output of 3 electrodes on different angles of APTN based prosthetic arm. Comparison of d) *V*_B_/*V*_F_, e) *V*_B_/*V*_D_, f) *V*_D_/*V*_B_. g) Digital response to APTN based prosthetic arm under different sliding force. h) The table shows the related locations and the controlled fingers of robotic hand when the finger sliding across on APTN based prosthetic arm. i) Change in the voltage when touching different segments. j) The photos of the robotic hand gestures corresponding to touch the seven different locations.

Tactile sensing is required for the dexterous manipulation of objects in prosthetic or robotic applications. Almost all of the former research focused on signals of the force amplitude. Here, we propose a prosthetic arm, inspired by a human arm (**Figure** **6**a), based on the APTN and its capability of measuring and discriminating in real‐time stimuli locations. A 3D printed mold with an arm‐shaped structure was designed as shown in Figure S8 (Supporting Information). A driver circuit was also used to communicate the APTN based prosthetic arm with a computer. The APTN based prosthetic arm formed by three bottom electrodes (*E*_b_, *E*_d_, *E*_f_) embedded into silicone rubber (Figure 6b). The gradient of APTN is also chosen to be tan *α* = 0.3. The length of the demonstrated prosthetic arm in this work is 5 cm. The grid layer is also deposited on the prosthetic arm by 3D printing. On the top view (Figure 6c), the angle between every two electrodes is 120°. As the working mechanism of the APTN is based on a single‐electrode TENG, the output signal is only generated on the operated electrode for sliding on individual electrode locations. However, when a stimulus is on the common area between two electrodes, the potential on both of the two adjacent electrodes will be induced. Thus, the locations between the two electrodes can be also detected. The electric potential on the electrode of the APTN based prosthetic arm decreases dramatically with increasing the thickness of silicone rubber of prosthetic arm. Eighteen locations could be discriminated by using three electrodes in this prosthetic arm. The connected driver circuit could record the peak voltage of the prosthetic arm. Figure 6d–f shows the peak voltage ratios of *E*_B_, *E*_D_, *E*_F_ (i.e., *V*_B_/*V*_F_, *V*_F_/*V*_D_, *V*_D_/*V*_B_) from point 1 to 18, respectively. As shown in Figure 6d, in terms of the same operated electrode *E*_B_ (sliding on 0° surface of prosthetic arm from top to bottom), the absolute magnitude of peak voltages of the *E*_B_ decreases. The related peak voltage ratio *V*_B_/*V*_F_ decreases from 153 to 62, which has the same tendency as the peak voltage of *E*_B._ This is because the peak voltage of *E*_F_ keeping nearly 0 V. The *V*_B_/*V*_F_ decreases from 0.8, 0.7, to 0.6 when sliding on the 60° surface of a prosthetic arm, indicating that the stimulation location is between *E*_B_ and *E*_F._ It also can be observed that the *V*_B_/*V*_F_ decreases from 78, 53, to 32 when sliding on the 300° surface of the prosthetic arm. Similarly, the peak voltage ratio *V*_F_/*V*_D_ is shown in Figure 6e. The value of *V*_F_/*V*_D_ decreases from 97, 70, to 40 when sliding on the 120° surface of the prosthetic arm. When the *V*_F_/*V*_D_ falling from 1, 0.9, to 0.6, the sliding location is between *E*_D_ and *E*_F_ (180°)_._ In Figure 6e, the value of *V*_D_/*V*_B_ decreases from 79, 59, to 36 when sliding on the 240° surface of the prosthetic arm. By analysing the different peak voltage ratios, the sliding locations in which angles can be recognized. After that, the peak voltage was compared, and the location was discriminated. In order to check the reliability of the prosthetic arm, the tests are conducted with *E*_B_ under different forces as shown in Figure 6g. Four groups of experiments are carried out under different forces. It can be seen that with the increase of force, the peak voltage of the *E*_B_ increases when sliding on 0° surface of prosthetic arm. However, the magnitude of the sliding force ranging from 1 to 4 N has a negligible effect on location recognition. This is because the increase of peak voltage following distance increasing is much more significant than the force increasing. Figure S9 (Supporting Information) illustrates the peak voltage values of sliding locations on three electrodes (location 1, 2, 3, 7, 8, 9, 13, 14, 15). The peak voltage of the different electrodes with the same distance between the top of the prosthetic arm and stimulation location is different because of some surface impurities and nonuniformity of silicone rubber. However, these differences do not influence the recognition of sliding locations as there's bigger differences in peak voltages when sliding on different locations. Here the APTN based prosthetic arm is further explored to control the robotic hands by detecting the location of touching. The robotic hand only has one joint at each finger, each one of the locations will control one or two joints of the robotic hand. The output voltage from APTN based prosthetic arm is recorded and processed to control the movement of the robotic hand. Figure 6h shows the related locations and the controlled fingers of robotic hand when the finger sliding across on APTN based prosthetic arm. The detailed voltage ratios *V*_B_/*V*_F_, *V*_F_/*V*_D_, and *V*_D_/*V*_B_ are also shown. Figure 6i shows the voltage signal of sliding locations (location 4, 5, 6, 10, 11, 12, 16, 17, 18). Figure 6i demonstrated the corresponding response gesture of the robotic hand. The output voltage with different magnitude of the different locations controls robotic hand. Sliding the locations 4, 5, 6, 10, 11, 12 (Figure 6j i–vi) controls only one finger bends. The location 16 (Figure 6j vii) controls two fingers to make a gesture “Yeah.. Therefore, the APTN based prosthetic arm has a great potential in real‐time control of the robotic hand to make gesture for diversified applications.

## 3 Conclusion

In summary, we propose a single‐electrode APTN here by constructing the dielectric layer with gradient thickness, and the tailorable property is coupled in it with shape variable and application reliable. The output of APTN is proved mainly depending on dielectric layer thickness after eliminating the influence of force and interlayer distance. By transmitting the response output signal to the driver circuit and analyzing the magnitude, the APTN could mimic the main functions of the biological sensory and nervous system to recognize the location of mechanical stimulation and transmitting mechanosensitive signal. When the APTN is tailored into an L‐shape, it exhibits superior advantages of single‐electrode output with the best gradient of tan *α* = 0.3 for 2D mechanical stimulation detection and control in tic‐tac‐toe game scenario. Moreover, analogous to the function of a biological interneuron in the spinal cord, when the APTN was designed as a prosthetic arm, it transmits and recognizes one location at a time. Then, this artificial sensory and nervous system identifies the location information about mechanical stimulation. This APTN has the potential to become a fundamental component in neuromorphic systems, human–machine interface, and artificial limb. The proposed artificial sensory and nervous system mimicking biological structures and function presents promising technology for the development of neuromorphic devices and artificial systems in the future.

## 4 Experimental Section

##### Fabrication of the APTN

A 3D‐printer (Anycubic 4Max Pro) was used to print 3D molds for preparing the single‐electrode TENG based APTN. The thicker edge of the dielectric layer of APTN will be changed from 0.5, 1, 2, 3, to 4 cm, while the thinner film keeps 0 cm. In other words, the gradient of dielectric layer thickness sets from tan *α* = 0.05, 0.1, 0.2, 0.3, to 0.4. The silicone rubber (Eco flex 00–50, Smooth‐on Inc.) was used as the dielectric material of APTN and the width of the devices keeps 2 cm. After curing, 5 devices with different gradient thickness was fabricated. For comparison, the device with a constant thickness of 0.5 cm was also fabricated. The mixture of PDMS (ratio: 10:1, sylgard 184, Dow Corning) was also poured into the mold with a constant thickness of 0.5 cm. Then a flexible conductive fabric was covered on silicone rubber or PDMS after it precuring with 15 min. The L‐shaped APTN and APTN based prosthetic arm were also fabricated by pouring silicone rubber in 3D printed molds which are designed, respectively. The gradient thicknesses of dielectric layers of L‐shaped and APTN based prosthetic arm are all tan *α* = 0.3.

##### Characterizations and Measurements

The short‐circuit current density of the S‐TENG were measured by a Stanford low‐noise current preamplifier (Model SR570). A digital oscilloscope (DS4052, RIGOL) was used to test the electric output voltage of the APTN. The resistance of the oscilloscope is 100 MΩ. The morphology of the surface of APTN was measured by field emission scanning electron microscopy (FESEM FEI Verios460). A force gauge testing system (Mecmesin, MultiTest 2.5‐i) is used for the test. The force gauge testing system is mainly composed of a fixed platform and a moving stage. Distance between the moving stage and fixed platform, force magnitude and moving speed of the moving stage can be preset. The moving stage moves toward the APTN in the preset speed until force reaches the presetting. Then the moving stage moves apart from the APTN with the presetting speed to the original position and begins next cycle.

##### Statistical Analysis

Each point data are the average of three peak currents or voltages. In addition, the standard deviations with the three peak currents or voltages are used as an indicator of statistical significance and reproducibility. Statistics were performed using the software Origin (Origin lab Corporation, USA).

## Conflict of Interest

The authors declare no conflict of interest.

## Supporting information

Supporting Information

## Data Availability

Data available on request from the authors: The data that support the findings of this study are available from the corresponding author upon reasonable request.
