# Supplementary material for: All in One, Self‐Powered Bionic Artificial Nerve Based on a Triboelectric Nanogenerator
Source: Adv Sci (Weinh). 2021 May 3;8(12):2004727. doi: 10.1002/advs.202004727 (PMC8224437; doi:10.1002/advs.202004727)
Supplement: Supplementary file 1 — Supporting Information [file ADVS-8-2004727-s001.pdf]

© 2021 Wiley-VCH GmbH

ADVANCED SCIENCE
Open Access

Supporting Information

for *Adv. Sci.,* DOI: 10.1002/advs.202004727

All in One, Self-powered Bionic Artificial Nerve Based on Triboelectric Nanogenerator

*Qian Zhang, Zixuan Zhang, Qijie Liang, Qiongfeng Shi, Minglu Zhu, and Chengkuo Lee**

Supporting Information

All in One, Self-powered Bionic Artificial Nerve Based on Triboelectric Nanogenerator

Qian Zhang[1,2], Zixuan Zhang[1,2,3], Qijie Liang[4], Qiongfeng Shi[1,2,3,5], Minglu Zhu[1,2,3,5], and Chengkuo Lee[1,2,3,5,6]*

[1] Department of Electrical and Computer Engineering, National University of Singapore, 4 Engineering Drive 3, Singapore 117576, Singapore

[2] Center for Intelligent Sensors and MEMS (CISM), National University of Singapore, 5 Engineering Drive 1, Singapore 117608, Singapore

[3] National University of Singapore Suzhou Research Institute (NUSRI), Suzhou Industrial Park, Suzhou 215123, China

[4] Department of Physics, National University of Singapore, 2 Science Drive 3, Singapore 117551, Singapore.

[5] Singapore Institute of Manufacturing Technology and National University of Singapore (SIMTech-NUS) Joint Lab on Large-area Flexible Hybrid Electronics, National University of Singapore, 4 Engineering Drive 3, Singapore 117576, Singapore

[6] NUS Graduate School for Integrative Science and Engineering (NGS), National University of Singapore, Singapore 117456, Singapore

* Corresponding author: elelc@nus.edu.sg (C.L.)

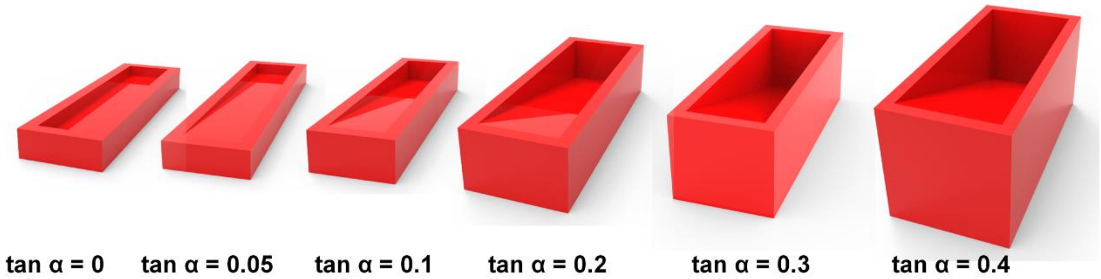

Fig. S1 | Moulds to fabricate the APTN with different gradient (tan α=0, 0.05, 0.1, 0.2, 0.3, 0.4)

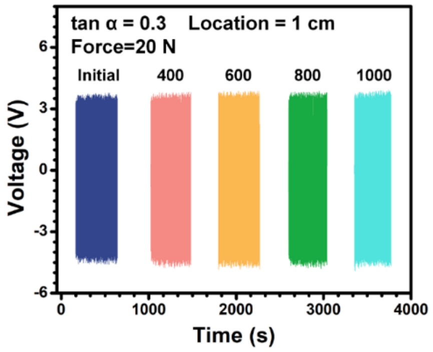

Fig. S2 | The stability test for the APTN with continuous touching on surface of the APTN by a force gauge stage for 1000 cycles.

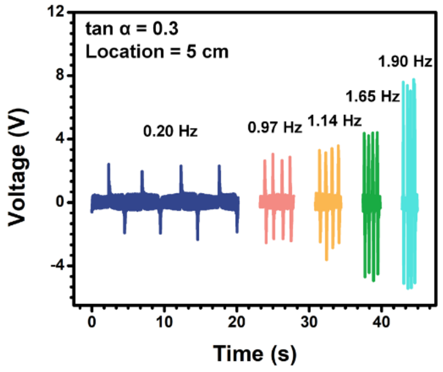

Fig. S3 | Voltage responses of APTN at different frequencies.

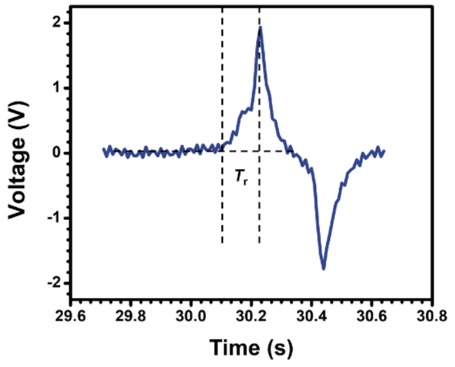

Fig. S4 | The enlarged view of the response of APTN with 2 cm grids, which renders a response time of 130 ms.

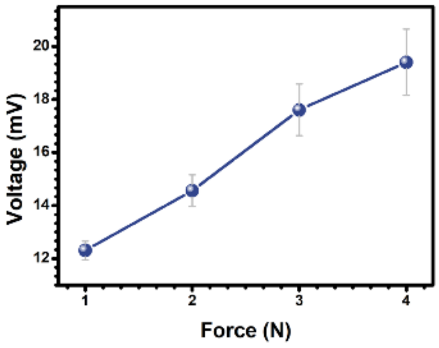

Fig. S5 | Relationship of output peak voltage of the force sensor and the applied force between 1 N to 4 N.

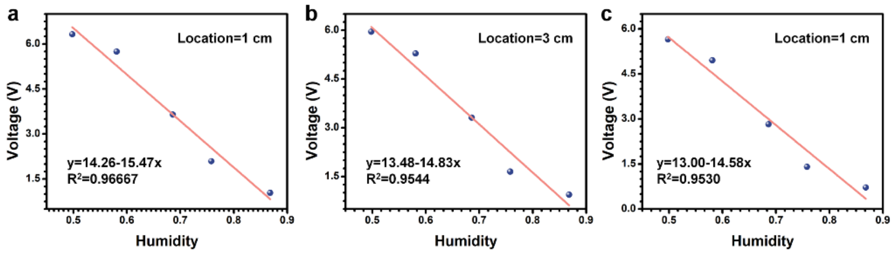

Fig. S6 | The output voltage of the APTN with a location of a) 1 cm, b) 3 cm and c) 5 cm as a function of humidity

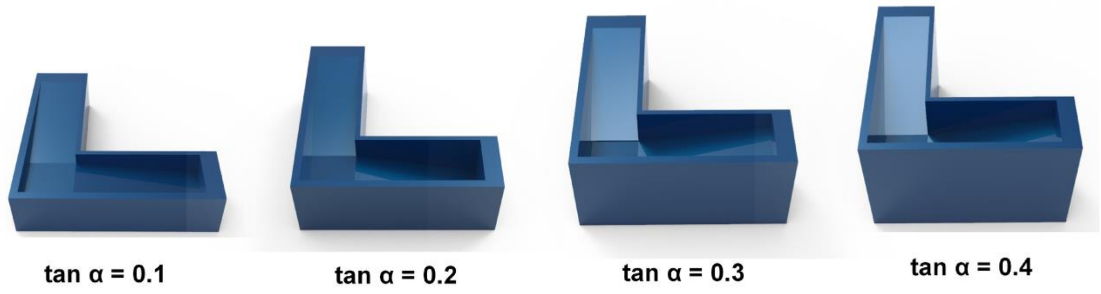

Fig. S7 | Moulds to fabricate the L-shaped APTN with different gradient (tan α= 0.1, 0.2, 0.3, 0.4).

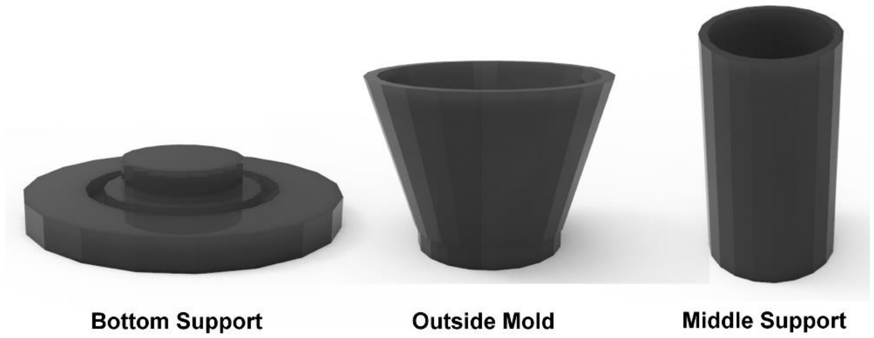

Fig. S8 | Moulds to fabricate the APTN based prosthetic arm with a gradient of tan α=0.3.

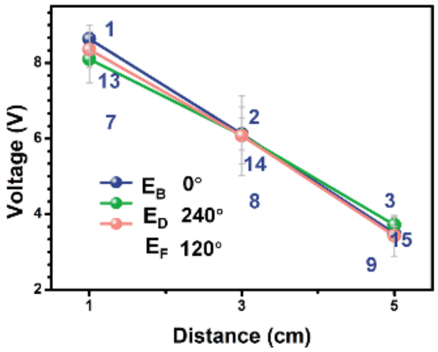

Fig. S9 | Digital voltage of three electrodes when a finger sliding on the relevant grid.
